# Chemical Characterization of *Enteromorpha prolifera* Extract Obtained by Enzyme-Assisted Extraction and Its Influence on the Metabolic Activity of Caco-2

**DOI:** 10.3390/ijms18030479

**Published:** 2017-02-23

**Authors:** Izabela Michalak, Agnieszka Dmytryk, Agnieszka Śmieszek, Krzysztof Marycz

**Affiliations:** 1 Department of Advanced Material Technologies, Wrocław University of Science and Technology, Smoluchowskiego 25 St., 50–372 Wrocław, Poland; agnieszka.dmytryk@pwr.edu.pl; 2 Scanning Electron Microscopy Laboratory, Wrocław University of Environmental and Life Science, Kożuchowska 5B St., 50–631 Wrocław, Poland; agnieszka.smieszek@up.wroc.pl (A.Ś.); krzysztof.marycz@up.wroc.pl (K.M.)

**Keywords:** algae, cellulase, enzymatic extract, elemental composition, polyphenols, cytotoxicity

## Abstract

The green seaweed *Enteromorpha prolifera* was used as a feedstock for the production of enzymatic hydrolysate using cellulase. The selection of the conditions for enzymatic hydrolysis of the biomass was carried out for different enzyme doses and incubation periods. The obtained extract was examined in terms of its multielemental composition, content of polyphenols and antibacterial properties (tested against *Escherichia coli* and *Staphylococcus aureus*). Additionally, its influence on the metabolic activity of human colon epithelial cells (Caco-2) was analyzed. The tested concentrations of extract using an in vitro model were 62.5, 125, 250, 500, 1000 and 2000 µg/mL. The hydrolysis yield in the most suitable experimental conditions (8-h process and 50 and 100 µL of cellulase) was 36%. Micro- and macroelements were poorly extracted from the algal biomass. Total phenolic content was 55 mg of gallic acid equivalent per 100 g of dry mass of extract. The cytotoxic effect of extracts, related to the inhibition of the metabolic activity of Caco-2, was noted only after 24 h. In turn, cultures of Caco-2 propagated with extracts for 72 h were characterized by significantly elevated metabolism (the concentration of extracts ranged from 62.5 to 1000 µg/mL, *p* < 0.05). Obtained results indicated the high biological activity of the prepared extracts; however, the observed effects did not occur in a dose-dependent manner.

## 1. Introduction

**Figure 1:**
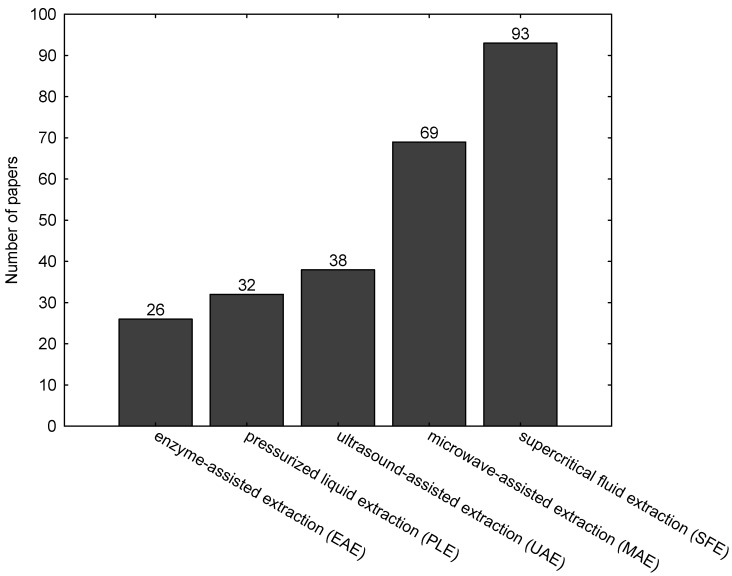
The number of scientific papers on extraction techniques of algae [2].

Different extraction techniques—traditional such as solvent extraction and novel such as supercritical fluid extraction (SFE), microwave-assisted extraction (MAE), ultrasound-assisted extraction (UAE), enzyme-assisted extraction (EAE) and pressurized liquid extraction (PLE)—are used to extract biologically active compounds from algae [1]. Among these techniques, the least scientific attention is paid to the enzymatic extraction of algae (Figure 1).

The accessibility of active compounds from algae can be limited due to the composition of the algal cell wall. The high content of various cell wall polysaccharides, especially cellulose, can limit the isolation of active compounds. Therefore, the enzymatic degradation of cell wall polymers is becoming an attractive alternative to chemical and mechanical processes [3]. Degradation of seaweed tissues helps release a variety of intracellular bioactive compounds [4,5]. Enzyme-assisted extraction is considered safe for labile active compounds such as phenolics [6].

Enzyme-assisted extraction is known to be an eco-friendly and nontoxic (without the use of organic solvents or other toxic chemicals) technique. Therefore, EAE has an advantage over traditional extraction techniques that use organic solvents. There are strict regulations for the use of organic solvents, for example in the food industry [5]. Additionally, the high extraction yield, moderate extraction conditions, and a lack of barriers such as water solubility and the insolubility of bioactive compounds are beneficial [1,7,8]. The main disadvantage of this method is the high cost of the enzymes [3,9,10]. Liang et al. (2012) indicated that the immobilization of enzymes can make them more economical since it facilitates the separation of enzyme from the product and its reuse [10]. In EAE, common enzymes that are used in the food industry (e.g., neutral and alkaline protease, α-amylase, cellulase and pepsin) can be used [3,8,11]. They are obtained by the fermentation of bacteria or fungi [11].

For the efficient extraction of compounds, the selection of appropriate enzymes and optimum extraction conditions is necessary. Wijesinghe and Jeon (2012) presented the optimum reaction conditions (pH and temperature) of the commonly used enzymes (Viscozyme, Celluclast, AMG, Termamyl, Ultraflo, Carrageenase, Agarase, Xylanase, Cellulase, Protamex, Kojizyme, Neutrase, Flavourzyme, Alcalase, Umamizyme) in the hydrolysis of seaweed cell walls [3]. Table 1 presents examples of the EAE of different biologically active compounds from macroalgae. This method is mainly used for the isolation of polysaccharides, phenols and pigments due to their water-soluble character [12,13]. The extracted compounds (especially polysaccharides and phenols) are known to possess antibacterial, antioxidative, anti-inflammatory, antitumor and antiviral properties [3,11,13].

In the present paper, EAE was performed using the enzyme cellulase and a green macroalga (*Enteromorpha prolifera*) as a substrate. This alga was chosen because its proliferation in water reservoirs (e.g., the Baltic Sea) constitutes a serious problem. Nowadays, the algae are mainly spreading on agricultural or composted land [22]. We propose the production of an algal extract with valuable properties. The biomass of *E. prolifera* is known to be a rich source of proteins, essential amino acids, minerals, fatty acids (including polyunsaturated fatty acids), vitamins, fibre and carbohydrates [23,24]. Taking into account the composition of the cell wall of *Enteromorpha intestinalis* (polysaccharides make up 63% of wall dry weight and incorporate glucose, galactose, xylose, rhamnose and glucuronic acid residues [25]), cellulase was chosen for the hydrolysis. The best conditions for the enzymatic hydrolysis were determined. The obtained algal extract was examined in terms of its multielemental composition, polyphenols content and antibacterial properties (gram-negative and gram-positive bacterial strains; *Escherichia coli* and *Staphylococcus aureus*, respectively). Additionally, we determined the biological proprieties of obtained extracts, namely cytotoxicity. Hence, the influence of enzymatic extracts on the metabolic activity of human colon epithelial cells (Caco-2) was analyzed. The Caco-2 cell line was chosen for this purpose, due to the morphological and biochemical characteristics of its cultures, which allows us to consider these cells as an established in vitro model for intestinal metabolism analysis [26].

## 2. Results and Discussion

### 2.1. Multielemental Composition of Algal Biomass, Enzymatic Extract and Post-Extraction Residue

In the literature, there is no evident interest in the elemental composition of algal extracts produced by different extraction techniques, especially by enzymatic hydrolysis. The composition of the raw algal biomass, algal extract obtained by EAE and post-extraction residue is presented in Table 2. Micro- and macroelements were poorly extracted from the biomass of *E. prolifera* using cellulase-assisted extraction. The enzymatic extract contained mainly light metal ions (Na extraction percentage (EP): 62%, Ca EP: 2.3%, K EP: 1.6%, Mg EP: 2.4%), as well as other elements like S EP: 1.6%, Fe EP: 0.71% and P EP: 1.3%. Among extracted microelements we can also distinguish Mn and Zn. Most of the elements remained in the post-extraction residue. The results obtained in the present study were compared with the multielemental composition of extracts produced from green Baltic macroalgae using SFE and MAE. Generally, for most of the elements, the extraction percentage from the raw biomass was lower than 10%, with the exception of Na for EAE; Cu, Mg, Na and S for SFE [27]; and K, Mg and Na for MAE [28].

### 2.2. Enzyme-Assisted Extraction of Algae

The enzymatic hydrolysis of the biomass, including algae, depends on several physicochemical factors starting with the process conditions (pH, temperature, time), through the type of solvent (reaction medium), ending with the ratio of substrate to enzyme and the enzyme concentration [3]. In order to limit the number of combinations, we decided to adapt variables from methodologies described by Heo et al. (2003) [29] (the most commonly used), Ghose (1987) [30] and Ahn et al. (2012) [21]. Finally, an experiment on EAE of *E. prolifera* was performed in 21 variants—including 20 at a whole range of Celluclast 1.5 doses. The selection of an appropriate extraction system was attempted since neither reference works nor reports following the methodology of Heo research group [5,31,32] included an optimization procedure or the results obtained thereby. Such an approach was, on the other hand, successfully applied to EAE of plant material—*Glycine max* and *Dimocarpus longan* [33,34]. Trivedi et al. (2013) elaborated guidelines for the optimization of bioethanol production from the green macroalga *Ulva fasciata* [20]. In each of those works, process efficiency was investigated based on the 3,5-dinitrosalicylic acid (DNS) assay for reducing sugars and the variant providing the highest saccharification yield was preferably chosen for the target application. The same criterion was adopted for enzymatic hydrolysis of *E. prolifera* biomass. Since EAE was proved a safe method for the isolation of susceptible compounds including phenolics from algae biomass, compared to conventional solvent processes [3,35,36,37], the content of phenolic compounds (see Section 2.3) and biological activity indicators (see Section 2.4 and Section 2.5) were verified only in the extract obtained under the most suitable conditions.

The concentration of glucose equivalent in EAE proved that biomass hydrolysis preferably involved cellulase solution rather than the solvent itself, since no released reducing sugars were detected in the untreated samples. The observed degradation of the algal cell wall differed as both enzyme dose and extraction time were taken into account (Table 3). At a given control point, one could note similar results (Δ < 2.5%, on average) within pairs of hydrolytic treatments: 10–20 μL and 50–100 μL of Celluclast 1.5. The latter provided more efficient biomass saccharification. The lowest reducing sugars release (RRS) values determined at 5 and 9.99 × 10*^−^*^2^% enzyme concentrations were similar to the highest means obtained for systems mediated with cellulase at 1 and 2 × 10*^−^*^2^%.

For each treatment examined, a significant increase in the extraction was achieved at the second control point. Statistical analysis showed the glucose equivalents released after 8-h processing at 50 μL and 100 μL enzyme doses to be significantly higher as compared to other dose- and time-related variants. A multitude of differences between entries—even below 10%—indicated as statistically significant might result from a small number of cases, and thus general trends were considered. Outlying decrease in measured values—such as low RRS at the third and fifth control points (all systems and 10/20 μL-mediated systems, respectively)—occurred in experiments on the extraction of natural materials [20]. Yet, they were excluded from further discussion.

The glucose equivalent determined at the first control point was the lowest and, simultaneously, different at *p* < 0.05 than the means at the second control point, regardless of the treatment performed. In the case of 50/100 μL-mediated systems, statistically significant differences between the results after 6-, 12- and 24-h extraction were also confirmed. The biomass hydrolysis by 50 and 100 μL of Celluclast 1.5 did not differ significantly after subsequent processing periods beginning from the second control point, and the similarity of the saccharification efficiency within two groups of operational times of eight, 12 and 24 h or 10, 12 and 24 h, respectively, was proven. No increase in RRS level corresponded to the end of the process. The enzymatic degradation was, on the other hand, unlikely to stop earlier as the significantly different effects of 6- and 8-h treatment indicated an ongoing reaction at that interval.

The results of glucose assay were confirmed by changes in enzyme activity (Table 4) leading to a 72%–76% decrease, about half of which occurred between the first and third control points. Relative activity below 100% reflected an apparent weakening of saccharification, and thus verified 8 h as the appropriate end time of the process since a statistically significant difference in the mean values (12%) was observed. Although a general trend of catalytic capacity lost was the same for each system, cellulase showed slightly higher stability at 50 and 100 μL doses—which differed significantly at *p* < 0.01 from the 10 μL-mediated system.

Under the experimental conditions, 5 and 9.99 × 10*^−^*^2^% solution of cellulase provided the highest total activity, differing significantly from treatments at lower concentrations. At the same time, absolute enzyme activity increased as the enzyme dose decreased. The absolute activity of Celluclast 1.5 in the examined systems differed from the value declared by the producer (endoglucanase activity of 700 U/g (approximately 843 U/mL)), as it would vary by dozens to thousands of U per enzyme unit dose depending on the substrate type [38]. Based on the negative dose-dependent correlation of absolute and total activity, it was considered that the reaction involved a determined pool of enzyme particles and further increasing the Celluclast 1.5 dose might not substantially enhance extract production. Therefore, no additional treatment was evaluated. Taking the obtained results into account, biomass hydrolysis seemed to be the most efficient after 8-h processing and at 50 and 100 µL of Celluclast 1.5. Since both cellulase concentrations showed almost equal hydrolytic capability, 5 × 10*^−^*^2^% was chosen for further investigation.

The selected system was compared to a variant performed in reduced volume of reaction medium per biomass unit weight. The use of 1:50 (*m/v*) biomass:medium ratio during 8-h hydrolysis enabled us to obtain 1.26 ± 0.01 mg/mL of glucose equivalent at enzyme total activity 1.46 ± 0.01 U. The efficiency of both variants was similar—differing by approximately 5%–6%, yet significantly in the case of enzyme activity. Slightly higher values of parameters led to the selection of the system with doubled biomass content as the best among those examined.

#### Extraction Yield

Enzyme-assisted extraction allows for successful production of water-soluble materials from seaweeds with a relatively high yield of around 50% [5]. This technique is known to improve the extraction efficiency of bioactive compounds when compared to aqueous extraction [16]. In the present work, the extraction yield of *E. prolifera* estimated for the chosen best variant (8-h extraction, 1 g:50 mL biomass:buffer ratio and 5 × 10*^−^*^2^% solution of enzyme) was 36%. Similar results were obtained for *Codium fragile* (a green seaweed), where the extraction yield with cellulase was 47.8 ± 3.5%, which was significantly higher than for the control (water blank, 22.4% ± 1.9%) [16]. These results are also in agreement with those obtained for the enzymatic extraction of brown macroalgae, for which yield ranged from 15% to 41% depending on the seaweed species [29]. Siriwardhana et al. (2004) found that the extraction yield from brown macroalga *Hizikia fusiformis* using carbohydrase–Celluclast was about 35% [12].

The extraction yield is mainly influenced by time, pH and temperature of hydrolysis [14,15], as well as seaweed species [16]. Enzyme-assisted extraction can provide higher yields in comparison to conventional extraction methods that use organic solvents such as toluene, hexane, acetone, methanol, ethanol or their combinations. Most of these solvents are unsuitable for the production of food ingredients or nutraceuticals. Therefore, there is a necessity to remove them from the final extract at elevated temperatures, what can lead to partial degradation of the extracted bioactive compound [14].

Enzymes can be also used to pre-treat the algal biomass before the proper extraction in order to hydrolyse cell wall polysaccharides and provide high extraction yields [1,14]. Billakanti et al. (2013) pre-treated the biomass of *Undaria pinnatifida* with enzyme–alginate lyase to efficiently extract in the next step fucoxanthin and lipids using dimethyl ether and ethanol. In this combined extraction method, enzyme-digested *Undaria* after centrifugation (removal of water-soluble compounds) was used for the extraction of lipophilic compounds using organic solvents. The enzymatic digestion of cell wall polysaccharides assisted in releasing the lipid-soluble compounds from *Undaria* seaweed [14].

### 2.3. Total Phenolic Content

Since the biochemical composition of algae varies due to various factors, such as location, season, growth conditions and biomass density, the content of phenolic compounds might differ between representatives of the same species collected from various aquatic environments [39]. Polyphenols together with other bioactive compounds (e.g., pigments, lipids, vitamins, polysaccharides and proteins) can be responsible for the antioxidative activity of the enzymatic extract of seaweeds (including *E. prolifera*) [13,18]. Enzyme-assisted extraction is considered a safe method for the isolation of labile active compounds such as phenolics [6,33]. After digestion of algal biomass by carbohydrolase, seaweed extracts could lead to exposing more phenolic compounds [31].

*Enteromorpha prolifera* extraction with cellulase, performed in the most suitable system (8 h, 1 g:25 μL:50 mL biomass:enzyme:buffer ratio), provided 55 ± 3 mg of gallic acid equivalent per 100 g of dry mass of the extract. At the same time, hydrolysates from *E. prolifera* characterized in the work of Ahn et al. (2012) showed a total phenolic content at the level of 151 mg/100 g [21]. Hardouin et al. (2014) examined the total polyphenolic content in the extracts obtained from green macroalga *Ulva* sp. using different carbohydrases—cellulase, xylanase, β-glucanase and arabanase. Extract produced with β-glucanase had the highest phenolic content among the examined carbohydrases—410 mg/100 g—but lower than for extracts obtained with proteases, endopeptidase and endoprotease. The highest polyphenolic content was noted in endoprotease extract and was equal to 990 mg/100 g. Additionally, it was found that enzymatic hydrolysis is less effective for polyphenol recovery. It is rather a promising technique for the extraction of proteins, neutral sugars, uronic acids and sulphate groups [15]. Heo et al. (2003) showed that among the tested carbohydrases—including Viscozyme, Celluclast, AMG, Termamyl and Ultraflo—Celluclast hydrolysate from *Ecklonia cava* had especially higher radical scavenging activities than the commercial synthetic antioxidant, butylated hydroxytoluene (BHT). It is emphasized that there is a correlation between the scavenging of 2,2-diphenyl-1-picrylhydrazyl (DPPH) free radicals and phenolic compounds; radical scavenging activity increases with an increase in phenolic content [29]. In the future, besides the determination of total phenolic content in enzymatic extracts from *E. prolifera*, detailed antioxidative properties should be evaluated by measuring radical scavenging activity, superoxide anion scavenging activity, hydroxyl radical scavenging activity and hydrogen peroxide scavenging activity. This issue is crucial because nowadays there is increasing interest in natural antioxidants that can replace commercial synthetic antioxidants. Seaweeds are good candidates because they are natural sources of antioxidants that are needed in the body and can also be used in different food formulations in order to extend their storage time, as well as in the pharmaceutical industry as antiaging and anticarcinogenic natural bioactive compounds [5,18].

### 2.4. Antibacterial Activity of Algal Extracts

Due to the resistance of pathogenic bacteria to existing antibiotics, there is a need to search for new biologically active compounds that possess antibacterial properties. Algae are considered promising candidates in novel antibacterial drug discovery [40]. Generally, green macroalgae contain biologically active compounds (e.g., proteins, polyphenols, polysaccharides, pigments and polyunsaturated fatty acids) that have antibacterial properties [13]. Extraction through the application of enzymes, such as cellulases, has the potential to increase the safety of algal compounds with antibacterial properties [13,40].

The enzymatic extract of *E. prolifera* slightly inhibited the growth of the gram-positive bacteria *Staphylococcus aureus* (zone of inhibition 0.66 mm), whereas in the case of gram-negative bacteria *Escherichia coli* no inhibition zone was observed. For the control group treated with gentamycin, the zone of inhibition of *Staphylococcus aureus* was 5 mm and for *Escherichia coli* it was 4.8 mm. These data are in agreement with the results presented by Qiao (2010) [41]. The enzyme (cellulase and hemicellulase) extracts of the brown alga *Laminaria digitata* showed no antimicrobial activity towards *Escherichia coli* and *Staphylococcus aureus*. A slight inhibition (<2 mm) of enzymatic extracts from *Ascophyllum nodosum* was observed only towards *Staphylococcus aureus* for both enzymes [41]. In the present study, it was found that the enzyme cellulase was not suitable for the extraction of antibacterial compounds from green macroalgae.

### 2.5. The Influence of Enteromorpha prolifera Enzymatic Extracts on the Metabolic Activity of Human Colon Epithelial Cells (Caco-2)

**Figure 2:**
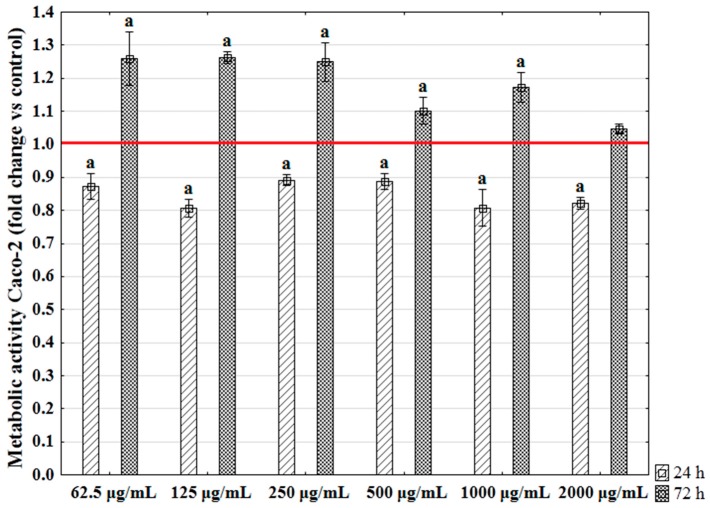
The influence of the *E. prolifera* extracts on Caco-2 metabolic activity. The statistically significant differences were tested at *p* level 0.05 (statistically significant difference marked as “a”). The normative value was determined based on a control culture’s metabolic activity (red line).

In this study, our purpose was to determine the influence of selected extract concentrations on the metabolic activity of Caco-2, which is associated with proliferative status. A decrease in metabolic status of Caco-2 cultures was observed after 24 h in culture, regardless of the concentration of the extract. Metabolic activity during the adaptive phase of Caco-2 growth was lowered by around 15% (±5%) in cultures with *E. prolifera*. A pro-proliferative effect of extracts on Caco-2 was noted at 72 h. The extract of *Enteromorpha* increased significantly metabolic activity of Caco-2 at concentrations of 62.5, 125, 250, 500 or 1000 µg/mL (Figure 2). The highest concentration tested, i.e., 2000 µg/mL, had no cytotoxic effect but did not promote Caco-2 metabolic activity either.

The obtained results revealed the cytotoxic effect of enzymatic extracts of *E. prolifera* at the initial phase of cell growth, i.e., after 24 h of culture incubation with the extracts. It was shown that the proliferation of Caco-2 began after the adaptive stage, which at a density of 10,000 per 1 cm^2^ may last 48 h [42]. Thus, we cannot exclude the fact that the observed cytotoxic effect is related to the elongation of the lag phase during the Caco-2 growth with the extract. Furthermore, the inhibition of Caco-2 metabolic activity was not strictly influenced by the extract concentration introduced to the culture environment. In turn, Caco-2 exposed for 72 h to the investigated extracts were characterized by increased metabolic activity. However, the dose-dependent manner of algae extract action was also ambiguous. According to the present results, the obtained extracts were free from the cytotoxic effects toward Caco-2 and therefore may have importance as a food component [43]. Algal extracts obtained using the EAE method were also not toxic toward other epithelial cell lines, e.g., VERO [11,44]. Typically, the extracts prepared using standard methods, e.g., organic or water, are cytotoxic at a very low concentration of algal biomass and are considered as potential anticancer agents [45,46]. In turn, the algal extracts obtained using the EAE method are well-tolerated by mammalian cells even at high concentrations [11,44]. However, the mechanism of the cytotoxic effect of extracts observed after 24 h of Caco-2 propagation should be further examined, because it may also be associated with the bioavailability of compounds derived from obtained extracts, as well as with their absorption kinetics [47].

## 3. Materials and Methods

### 3.1. Algal Biomass

Free-floating thalli of green marine species *E. prolifera* were collected by hand from the Baltic Sea water in Rewal, Poland (54°04′58.1″ N 15°00′36.8″ E) in June 2015. The macroalgae were promptly transported to the laboratory and stored at temperature −20 °C prior to the extraction. Just before the extraction process, the material was defrosted and rinsed with fresh water three times to remove contaminants, including salt, sand, small insects and epiphytes. Algal biomass was dried to constant weight for one week in the open air and then ground using a hand blender and sifted with a sieve to obtain a fine fraction with particle size <500 μm.

### 3.2. Multielemental Analysis of Algal Biomass

The multielemental analysis of the raw biomass of *E. prolifera* and post-extraction residues was performed using the Inductively Coupled Plasma–Optical Emission Spectrometry (ICP–OES) technique. The analyses were carried out on ICP–OES type iCAP 6500 Axial and Radial Vista (Thermo Scientific, Waltham, MA, USA), according to international standards, such as PN-EN ISO/IEC 17025:2005—General requirements for the competence of testing and calibration laboratories. Before multielemental analysis, the samples (about 0.5 g of dry mass) underwent mineralization with 5 mL of 69% HNO_3_ (Merck KGaA, Darmstadt, Germany) in Teflon bombs using a microwave oven—Milestone Start D (Milestone S.r.l., Sorisole, Italy).

### 3.3. Selection of Hydrolysis System for Preparation of Enzymatic Extracts

#### 3.3.1. Hydrolysis Experiments

Production of *E. prolifera* extract involved commercially available cellulase from the fungus *Trichoderma reesei* ATCC 26921—Celluclast 1.5 (synonym 1,4-(1,3:1,4)-β-d-glucan 4-glucano-hydrolase; Sigma-Aldrich Chemie GmBh, Schnelldorf, Germany). The extraction procedure was based on an International Union of Pure and Applied Chemistry (IUPAC) report on cellulase activity measurements [30] and the methodology described by Heo et al. (2003) [19,29]. The latter was previously adapted to the treatment of green macroalgae—including *Enteromorpha* species [31]. In the present work, different variants of the experiment were examined (Table 5). In addition, elements of a system for *E. prolifera* hydrolysis reported by Ahn et al. (2012) were also verified [21].

While the selection of the conditions of enzymatic hydrolysis included enzyme doses and incubation time, other process variables such as biomass sample weight, volume and pH of reaction medium, rotary speed and temperature were the same for each trial. The reaction medium was 0.05 M citrate buffer, prepared according to the recipe of Lillie (1948) using citric acid monohydrate and trisodium citrate dihydrate (both from Avantor Performance Materials Poland S.A., Gliwice, Poland) [48]. Hydrolysis started as the biomass was suspended in an enzyme–buffer mixture pre-incubated to the target temperature. In order to exclude the effect of solvent extraction on the studied process, an untreated sample was also investigated. Enzyme-assisted extraction trials were carried out in an air bath orbital shaker-incubator (model ES-20/60; Biosan, Riga, Latvia). A given enzyme dose was applied to one system, from which samples were collected at the time intervals shown in Table 5 until the process ended after 24 h.

Each time of consecutive sample collection was hereinafter called a control point. Post-extraction mixtures were separated from unhydrolyzed residues twice by centrifugation at 4500 rpm for 10 min (Heraeus Megafuge 40, rotor TX-750, Thermo Scientific, Waltham, MA, USA). The precipitate was weighed for evaluation of the process yield. At the same time, supernatants were eventually clarified by passing through filter paper (MN 615; Macherey-Nagel, Düren, Germany) and thus we obtained filtrates—referred to as final *E. prolifera* extracts—that were stored at −20 °C until use for measurement of multielemental, total phenolic content and both antibacterial and metabolic activity.

For the variant, which provided the most efficient extract production (monitored by the changes in enzyme capability to release reducing sugars from algal cell walls), the process was repeated in a buffer volume of 50 mL per algal powder unit weight. The reaction medium was adjusted to a total volume of 200 mL to facilitate sample collection. The last variant was applied to verify whether the substrate:medium ratio might positively influence the yield of the hydrolysis.

#### 3.3.2. Glucose Assay by 3,5-Dinitrosalicylic Acid Colorimetric Method

Released reducing sugars assay enables us to evaluate hydrolysis efficacy and hence cellulase activity [30,49]. The concentration of RRS, expressed as the d-glucose equivalent, was determined by a modified spectrophotometric method using 3,5-dinitrosalicylic acid (DNS) (Sigma–Aldrich, Alwar, India) [50], developed at the University of Maryland, College Park, MD, USA [51]. A composition of DNS reagent contained—besides 1% (*m/v*) DNS solution in water—sodium sulphite and sodium hydroxide at 0.5 and 10 g/L, respectively.

Since glucose measurements were performed several times during the hydrolysis to verify its progress, the collection of extract samples followed a 10-min pause in shaking during which the biomass sedimented. A sample with a volume of 500 µL was collected from the top of a given extract, diluted 18 times with 0.05 M citrate buffer (pH 4.8), and divided into three aliquots (replicates). For assay purposes, 3 mL of each replication was mixed with an equal volume of DNS reagent and then heated at 90 °C for 15 min to enable deep red-brown colour development. Afterwards, 1 mL of 40% potassium sodium tartrate (C_4_H_4_KNaO_6_; Rochelle salt, Avantor Performance Materials Poland S.A., Gliwice, Poland) was added to stabilize the colour and the obtained mixtures were cooled to room temperature in a cold water bath. Absorbance was measured with a spectrophotometer (50 UV-Vis Spectrophotometer, Varian Cary, Victoria, Australia) at 575 nm against a blank sample containing 0.05 M citrate buffer (pH 4.8) instead of the extract. Released reducing sugars concentration in extract samples was estimated based on a calibration plot for glucose as a standard.

The calibration curve for glucose (d-(+)-Glucose from Avantor Performance Materials Poland S.A. (Gliwice, Poland) was plotted for the concentration range of 1.8 × 10^−2^–1.8 mg/mL, which corresponded to 0.1–10 mM. Calibration samples were prepared by serial dilutions of 1.8 mg/mL glucose stock solution. For preparation of stock solution and dilution series, 0.05 M citrate buffer (pH 4.8) was used. The glucose concentration in calibration samples was measured according to the foregoing DNS colorimetric method. Each glucose solution underwent a 15-min incubation at 90 °C with 1% DNS reagent (3 mL:3 mL), which preceded the addition of 40% Rochelle salt (1 mL). After cooling samples to room temperature, spectrophotometric measurements were performed against a blank sample in which 0.05 M citrate buffer (pH 4.8) replaced glucose. Measurements were carried out in triplicate. The equation describing the obtained standard curve for glucose was as follows:

(1) y = 0.4557x−0.022 ,

where *y* is the absorbance and *x* is the glucose concentration (mg/mL). The *R*^2^ value was 0.998.

#### 3.3.3. Selection of Proper Hydrolysis System—Calculations

The most appropriate hydrolysis system was selected after the comparison of the concentration of glucose (Equations (2a) and (2b)), and both absolute—*A_Abs_* (Equations (4a)–(4c)) and relative enzyme activity—*A*_Rel_ (5), calculated on the basis of the calibration equation for RRS assay (1).

Released reducing sugars concentration as glucose equivalent in examined systems:

(2a) $$C_{RRS}=C_{Gl}=\;\frac{y-b}{a}\cdot D_{S}$$

(2b) $$C_{Gl}=39.5y+0.897,$$

where *C_Gl_* is the concentration of glucose (mg/mL), *y* is the average absorbance, *a* is the slope of the calibration curve (mL/mg), *b* is the *y*-intercept of the calibration curve and *D_S_* is the sample dilution.

Total activity of Celluclast 1.5 in examined systems:

(3a) $$A_{Tot}=\;\frac{C_{Gl}\cdot V_{Ex}}{M_{Gl}\cdot t}\;\cdot 1000\;$$

(3b) $$A_{Tot}=\;\frac{C_{Gl}\cdot \left(V_{Ex,0}-\left(k-1\right)\cdot V_{S}\right)}{M_{Gl}\cdot t}\cdot 1000\;$$

(3c) $$A_{Tot}≅555\left[\frac{\mathrm{mL}\cdot \mu \mathrm{mol}}{\mathrm{mg}}\right]\cdot \frac{C_{Gl}}{t}\;,$$

where *A_Tot_* is the total enzyme activity (U), *M*_Gl_ is the molar mass of glucose (mg/mmol), *t* is the control point (min), *k* is the ordinal number of a given control point, *V_Ex,_*_0_ and *V_Ex_*—are the extract volume at the beginning and a given time of the process, respectively (mL), *V*_S_ is the sample volume (mL), and *C_Gl_* is as in Equations (2a) and (2b).

Absolute activity of Celluclast 1.5 in examined systems:

(4a) $$A_{Abs}=\;\frac{A_{Tot}}{V_{En}}$$

(4b) $$A_{Abs}=\;\frac{A_{Tot}}{\left(V_{En,0}-\frac{\left(k-1\right)\cdot V_{S}}{D_{En}}\right)}$$

(4c) $$A_{Abs}=\frac{A_{Tot}}{\left(V_{En,0}-\frac{\left(k-1\right)\cdot 0.5\left[\mathrm{mL}\right]}{D_{En}}\right)}\;,$$

where *A_Abs_* is the absolute enzyme activity (U/mL), *A_Tot_* is the total enzyme activity at a given control point (U), *V_En,0_* and *V_En_* are the enzyme volume at the beginning and a given time of the process, respectively (mL), *D_En_* is the enzyme dilution in medium, and *k* and *V*_S_ are as in Equations (3a), (3b) and (3c).

Relative activity of Celluclast 1.5 in examined systems:

(5) $$A_{Rel}=\;\frac{A_{Tot,t}}{A_{Tot,6\;h}}\cdot 100\%,$$

where *A_Rel_* is the relative enzyme activity (%), *A_Tot,6 h_* is the total enzyme activity after 6-h hydrolysis (U) and *A_Tot,t_* is the total enzyme activity at subsequent control points (U).

### 3.4. Measurement of Extraction Yield

Yield of the extraction of seaweed was calculated as a quotient of dry weight of hydrolyzed filtrate over dry weight of the seaweed sample used [29].

### 3.5. Total Phenolic Content in Enzymatic Hydrolysates

Phenolic content was determined with a Folin–Ciocalteu reagent using a method described by Shetty et al. (1995) [52]. It was expressed as gallic acid equivalent (GAE). One millilitre of the final extract, obtained under the best conditions (8-h extraction, 1 g:50 mL biomass:buffer ratio and 5 × 10^−2^% solution of enzyme), was incubated with 1 mL of 95% ethanol (Avantor Performance Materials Poland S.A., Gliwice, Poland), 5 mL of deionized water and 0.5 mL of 50% Folin–Ciocalteu reagent (Merck KGaA, Darmstadt, Germany) at room temperature for 5 min. The obtained solution was then mixed with 1 mL of 5% Na_2_CO_3_ (Avantor Performance Materials Poland S.A., Gliwice, Poland) and allowed to react in the dark for 1 h. Absorbance was measured in triplicate at 725 nm against the aforementioned mixture without the extract. The content of phenolic compounds in the extract was estimated on the basis of a calibration plot for gallic acid as a standard.

The gallic acid calibration plot was prepared according to the procedure described by Shetty et al. (1995) [52]. A stock solution (1 g/L) of gallic acid (Sigma-Aldrich, Shanghai, China) in 0.05 M citrate buffer pH 4.8 was serially diluted with the same buffer to obtain calibration samples at a concentration range from 12.5 to 75 mg/L. The mixture (1:1:5:0.5, *v/v/v/v*) containing a given gallic acid dilution, 95% ethanol, deionized water and 50% Folin–Ciocalteu reagent was reacted for 5 min and then subjected to 1-h dark incubation with 5% Na_2_CO_3_. Afterwards, samples were measured spectrophotometrically against a blank sample to which gallic acid was not added. Measurements were performed in triplicate. The equation describing the obtained standard curve for gallic acid was as follows:

(6) y = 0.0015x + 0.0147 ,

where *y* is the absorbance and *x* is the phenol concentration (mg/L). The *R*^2^ value was 0.998.

According to the work of Ahn et al. (2012), total phenolic content is calculated as gallic acid equivalent per 100 g of extract dry mass [21]:

(7a) $$T=\frac{\left(y-b\right)\cdot V_{Ex}}{a\cdot m_{Ex}}\;\times \;100\;$$

(7b) $$T=13333\cdot \frac{\left(y-0.0147\right)}{m_{Ex}}\;,$$

where *T* is the total phenolic content (mg/100 g), *y* is the average absorbance, *a* is the slope of the calibration curve (L/mg), *b* is the *y*-intercept of the calibration curve, *V_Ex_* is the total volume of the extract solution (L) and *m_Ex_* is the dry mass of the extract (g).

### 3.6. Antibacterial Activity of Algal Extracts

The antibacterial activity of the algal extract was determined by the Kirby–Bauer disk diffusion method, as previously described [53]. Two bacterial strains, Gram-negative (*Escherichia coli*—ATCC 8739) and Gram-positive (*Staphylococcus aureus*—ATCC 25923), were examined. The bacterial inocula were grown overnight in lysogeny broth (LB), that contained tryptone, pancreatic-digested casein hydrolyzate (10 g), yeast extract (5 g) and NaCl (10 g). Final pH at 25 °C was 7. A small amount of bacteria (about 1–2 × 10^8^ colony forming unit (CFU) per mL) was taken using the inoculation loop. The bacterial inoculum was suspended in test tubes with a saline solution to obtain a suspension that matched the turbidity of a 0.5 McFarland standard. The diluted bacterial culture was placed on Tryptic Soy Agar (TSA) medium and spread throughout sterile Petri dishes using a sterile glass “L” rod. This formed the bacterial lawn. Tryptic Soy Agar was composed of tryptone (15 g), soytone—an enzymatic digest of soybean meal (5 g), sodium chloride (5 g) and agar (15 g). The final pH at 25 °C was 7. The paper discs with 5 mm diameter (prepared from Whatman No. 1 filter paper) were soaked with algal extract (obtained under the best experimental conditions: 8-h extraction, 1 g:50 mL biomass:buffer ratio and 5 × 10^−2^% solution of enzyme), dried at room temperature for 15 min and placed on Petri dishes with a TSA medium. The dishes were incubated for 20 h at 37 °C. Antibacterial activity was recorded by measuring the diameter of the zone of inhibition. Gentamicin (concentration 10 mg/mL) was used as a positive reference.

### 3.7. The Influence of Enteromorpha prolifera Enzymatic Extracts on the Metabolic Activity of Human Colon Epithelial Cells (Caco-2)

#### 3.7.1. Propagation of Cells/Cell Culture

The human colon epithelial cell line Caco-2 (ATCC HTB-37™, LGC Standards, Łomianki, Poland) was used in the experiment. Caco-2 cells were propagated in a complete growth medium (CGM), i.e., Dulbecco’s Modified Eagle’s Medium (DMEM; Sigma–Aldrich, Poznań, Poland) containing 4500 mg/L of glucose. The medium was supplemented with a 10% fetal bovine serum (FBS) (Thermo Fisher Scientific, Warsaw, Poland), a 2% 4-(2-hydroxyethyl)-1-piperazineethanesulfonic acid (HEPES) buffer (Sigma–Aldrich) and a 1% solution of minimum essential medium (MEM) non-essential amino acid (Thermo Fisher Scientific, Warsaw, Poland). Furthermore, 1% of the medium constituted antibiotics (gentamycin, penicillin, streptomycin and amphotericin B (Sigma-Aldrich, Poznań, Poland). After reaching 80% confluence, cultures were passaged using a trypsin– ethylenediaminetetraacetic acid (EDTA) solution (TrypLE, Thermo Fisher Scientific, Warsaw, Poland).

#### 3.7.2. Determination of Proliferative Activity—Alamar Blue Test

Caco-2 cells were seeded into 96-well plates at a concentration of 10,000 cells per well. Previous studies showed that this initial concentration allows us to maintain the shortest population doubling time for Caco-2 [42]. Cells were inoculated in 0.2 mL of culture medium. Enzymatic extracts of algae (obtained under the best experimental conditions: 8-h extraction, 1 g:50 mL biomass:buffer ratio and 5 × 10^−2^% solution of enzyme) were added to the Caco-2 culture when about 90% of the inoculated cells were adhered to the microplate. The following concentrations of extracts were tested: 62.5, 125, 250, 500, 1000, and 2000 µg/mL. In order to determine the exact effect of the algal extract on the metabolic activity of Caco-2 and eliminate the effect of cellulase, the following negative controls were used: (i) Caco-2 culture in CGM with proper addition of cellulase alone, and (ii) untreated cells propagated in CGM. The metabolic activity of Caco-2 in cultures with extract was assessed after 24 and 72 h. For this purpose, Alamar Blue (Resazurin-based assay, Sigma–Aldrich) was used as previously described [35]. The dye was added to the complete growth culture medium in an amount equal to 10% of the culture medium volume. The cultures were incubated with the reagent for two hours in the CO_2_ incubator. The supernatants were collected and transferred to a 96-well microplate reader (Spectrostar Nano, BMG Labtech, Ortenberg, Germany). The absorbance of the supernatants was measured spectrophotometrically at a wavelength of 600 nm for resazurin and 690 nm as a reference wavelength. Each measurement included a blank sample, containing the complete medium without cells. The proliferation factor (PF) was determined in order to describe the influence of the algal extract on Caco-2. The obtained values of PF represent the norm evaluated with regards to the negative controls of the experiment. The activity of cells in control cultures was established as equal to 1; therefore, PF values higher than 1 expressed an increase in cellular activity resulting from the algae extract addition, whereas values lower than 1 showed a reduction in the rate of cell proliferation.

### 3.8. Statistical Analysis

Statistical analysis was performed using Statistica 12.0 software (StatSoft Polska Sp. z o.o., Kraków, Poland). The significance of differences between the normative values determined for control and experimental cultures was evaluated using the paired Student’s *t*-test. The results were considered significant at *p* < 0.01 and 0.01 ≤ *p <* 0.05. All data are presented as mean ± standard deviation (SD).

## 4. Conclusions

Nowadays, there is a need to develop novel extraction techniques in order to isolate natural biologically active compounds that can be utilized in the cosmetic, pharmaceutical and food industry. Enzyme-assisted extraction, due to its efficiency and mild reactive conditions, can be an alternative to conventional extraction techniques that use organic solvents. Hydrolysates obtained by enzyme extraction are safe because they are solvent-free. In the present paper, we proposed to perform enzyme-assisted extraction with cellulase of a green macroalga (*E. prolifera*), since it is a promising candidate for a wide variety of biologically active compounds. The best extraction conditions were determined. They involved: 8-h extraction, 1 g:50 mL biomass:buffer ratio and 5 × 10^−2^% solution of enzyme. Extraction yield was equal to 36%. The enzymatic extract was tested for the concentration of elements, antibacterial and antioxidative properties, and its influence on the metabolic activity of Caco-2. It was shown that micro- and macroelements were poorly extracted from the algal biomass. Enzymatic extract possessed antioxidative properties—55 ± 3 mg of gallic acid equivalent per 100 g of dry mass of the extract. The tested enzyme, cellulase, was not suitable for the extraction of antibacterial compounds. Algal extract slightly inhibited the growth of *Staphylococcus aureus*. In the case of *Escherichia coli*, no inhibition zone was observed. The analysis of biological activity revealed that enzymatic extracts from *E. prolifera* did not show cytotoxic effect and promoted metabolic activity in cultures propagated for 72 h. The inhibition of Caco-2 metabolism, observed at 24 h of culture with extracts, should be investigated in greater detail, as it may reflect the bioavailability of the compounds derived from *E. prolifera*. Interestingly, the influence of enzymatic extracts of *E. prolifera* on Caco-2 did not depend on their concentration. Future detailing of the characteristics of enzymatic extracts from green macroalgae is needed in order to propose a safe product for use in the food, cosmetic and pharmaceutical industries.

## Figures and Tables

**Table 1:** Examples of the enzyme-assisted extraction of biologically active compounds from the biomass of macroalgae and their applications.

Enzyme	Alga	Extracted Compound	Method	Application	Reference
Neutrase 0.8 L (endoprotease—neutral *Bacillus amyloliquefaciens* protease)	*Sargassum coreanum* (B)	crude polysaccharide (fucose, galactose, and glucose)	50 g A, 5 g E, 0.2 M PB, pH 8, 50 °C, 12 h	inhibitory activities against cancer cell growth, induction of apoptosis in HL-60 tumor cells	[7]
Alginate lyase (combined extraction method: enzyme pre-treated *Undaria* after centrifugation was used to extract fucoxanthin and lipids using organic solvents)	*Undaria pinnatifida* (B)	fucoxanthin, lipids containing polyunsaturated fatty acid	0.05% (*v/w*) A, 10 mg/mL E, 0.1 M PB, pH 6.2, 37 °C, 2 h	possible alternative to ethanol for the extraction of fucoxanthin from *U. pinnatifida*	[14]
Carbohydrases (cellulase, xylanase, arabanase, β-glucanase); Proteases (endopeptidase, endoprotease)	*Ulva* sp. (G) *Sargassum muticum* (B) *Solieria chordalis* (R)	soluble protein, polyphenol, neutral sugars, uronic acids, sulphated polysaccharides	500 g A, 0.5% (*w/w*) E, 500 mL H_2_O, 50 °C, 5 h (example for *Ulva* sp.)	antiviral activity without cytotoxicity effect–tested Herpes simplex virus 1 (HSV-1)	[15]
Carbohydrase (cellulase, β-glucanase, Ultaflo (heat-stable multi-active β-glucanase)); Protease	*Chondrus crispus* (R) *Codium fragile* (G)	protein, neutral sugars, uronic acids, and sulfates	1 and 10 g A, 0.5% E, 200 mL H_2_O, 50 °C, 3 h	functional food and antiviral drug discovery—the enzymatic hydrolysates exhibited significant activity against HSV-1	[16]
Carbohydrases: Celluclast (β-glucanase), exo-1,4-α-d-glucosidase (AMG), Termantyl (heat-stable α-amylase), Ultaflo, Viscozyme (arabanase, cellulase, β-glucanase, hemicellulase and xyianase); Proteases: Alcalase (endoprotease), Protamex (endoprotease), Neutrase, Flavourzyme (endoprotease and exopeptidase activities), Kojizyme (endo/exopeptidase)	*Sargassum horneri* (B)		1 g A, 100 µL (or mg) E, 100 mL of buffer (AB for Viscozyme, AMG, Celluclast, for the rest—PB), 12 h	antioxidant activity (use in different food formulations and pharmaceutical industry)	[5,17,18]
Carbohydrases: Celluclast (β-glucanase), exo-1,4-α-d-glucosidase (AMG), Termantyl (heat-stable *α*-amylase), Ultaflo, Viscozyme (arabanase, cellulase, β-glucanase, hemicellulase and xyianase); Proteases: Alcalase (endoprotease), Protamex (endoprotease), Neutrase, Flavourzyme (endoprotease and exopeptidase activities), Kojizyme (endo/exopeptidase)	*Ecklonia cava* (B)	antioxidant compounds	1 g A, 100 µL (or mg) E, 100 mL of buffer (AB for Viscozyme, AMG, Celluclast, for the rest—PB), 12 h	antioxidant activity (use in different food formulations and pharmaceutical industry)	[19]
Viscozyme L (cellulase)	*Ulva fasciata* (G)	-	1 g A, 1%, 2% and 5% (*v/v*) E, 20 mL of sodium acetate buffer (pH 4.8), 45 °C, 42 h	production of bioethanol	[20]
Carbohydrases (amyloglucosidase 300 L (AMG), Celluclast 1.5 L FG, Dextrozyme, Maltogenase, Promozyme, Viscozyme L and Termamyl); Proteases (Alcalase 2.4 L FG, Flavourzyme 500 MG, Neutrase 0.8 L, Protamex)	*Enteromorpha prolifera* (G)	-	1 g A, 20 mg E, 60 mL of H_2_O, 8 h	antioxidant, anti-acetylcholinesterase and anti-inflammatory activity	[21]
Carbohydrases (Viscozyme, Celluclast, Termamyl and Ultraflo); Proteases (Protamex, Kojizyme, Neutrase, Flavourzyme and Alcalase)	*Hizikia fusiformis* (B)	antioxidant compounds—polyphenols	1 g A, 100 mL of distilled water, 5%—enzyme/substrate ratio, 3 days, final pH 7.0	antioxidant activity	[12]

A: Dried alga; E: Enzyme; PB: Phosphate buffer; AB: Acetate buffer; B: Brown macroalga; G: Green macroalga; R: Red macroalga.

**Table 2:** The multielemental composition of raw algal biomass, algal extract obtained by the enzyme-assisted extraction and post-extraction residue (*n* = 3).

Element	Wavelength (nm)	Algae before Extraction (mg/kg of Dry Mass (d.m.))	Algal Extract—EAE (mg/L)	Post-Extraction Residue of Algae (mg/kg of d.m.)	Algal Extract—SFE * (mg/L) [27]	Algal Extract—MAE ** (mg/L) [28]
As	188.980	<LLD	<LLD	<LLD	0.72 ± 0.09 (EP: 7.4%)	0.198 ± 0.025 (EP: 5.1%)
B	249.772	231 ± 35	4.69 ± 0.70 (EP: 2.0%)	37.2 ± 5.6	<LLD	4.74 ± 0.71 (EP: 4.8%)
Ba	455.403	26.9 ± 4.0	1.04 ± 0.16 (EP: 3.9%)	26.4 ± 4.0	-	-
Ca	315.887	16,540 ± 3308	381 ± 57 (EP: 7.4%)	9412 ± 1882	1060 ± 210 (EP: 7.4%)	365 ± 54 (EP: 0.9%)
Cd	228.802	0.260 ± 0.039	<LLD	0.010 ± 0.002	<LLD	0.001 ± 0.000 (EP: 0.14%)
Co	228.615	2.41 ± 0.36	0.035 ± 0.009 (EP: 1.6%)	1.49 ± 0.22	0.026 ± 0.004 (EP: 0.54%)	0.0135 ± 0.0034 (EP: 0.47%)
Cr	267.716	3.79 ± 0.57	0.022 ± 0.006 (EP: 0.58%)	3.65 ± 0.55	0.31 ± 0.05 (EP: 2.4%)	-
Cu	324.754	7.09 ± 1.06	0.23 ± 0.03 (EP: 3.2%)	6.50 ± 0.97	6.3 ± 0.9 (EP: 22%)	0.108 ± 0.016 (EP: 0.85%)
Fe	259.940	2440 ± 488	17.3 ± 2.6 (EP: 0.71%)	2114 ± 423	9.2 ± 1.4 (EP: 0.1%)	4.47 ± 0.70 (EP: 0.07%)
K	766.491	2705 ± 541	42.3 ± 6.3 (EP: 1.6%)	1293 ± 259	52 ± 8 (EP: 1.1%)	951 ± 142 (EP: 19%)
Mg	285.213	5993 ± 1199	142 ± 21 (EP: 2.4%)	5180 ± 1036	406 ± 61 (EP: 10%)	322 ± 48 (EP: 10%)
Mn	257.61	322 ± 48	7.09 ± 1.06 (EP: 2.2%)	281 ± 42	6.6 ± 1.0 (EP: 2.4%)	3.07 ± 0.46 (EP: 1.3%)
Na	588.995	2586 ± 517	1593 ± 319 (EP: 62%)	1887 ± 377	965 ± 145 (EP: 17%)	1250 ± 250 (EP: 20%)
Ni	231.604	9.01 ± 1.35	0.255 ± 0.038 (EP: 2.8%)	6.29 ± 0.94	0.27 ± 0.04 (EP: 3.0%)	0.132 ± 0.019 (EP: 2.5%)
P	213.618	1168 ± 234	15.1 ± 2.3 (EP: 1.3%)	923 ± 138	43 ± 6 (EP: 2.8%)	32.9 ± 4.9 (EP: 2.8%)
S	181.972	18,381 ± 3676	292 ± 44 (EP: 1.6%)	10,890 ± 2178	9300 ± 1900 (EP: 92%)	702 ± 105 (EP: 8.1%)
Si	251.611	668 ± 100	6.65 ± 0.99 (EP: 1.0%)	682 ± 102	-	11.9 ± 1.8 (EP: 1.3%)
Zn	213.857	227 ± 34	3.70 ± 0.56 (EP: 1.6%)	64.3 ± 9.6	5.2 ± 0.8 (EP: 3.0%)	0.169 ± 0.025 (EP: 0.26%)

* SFE: Supercritical fluid extraction of Baltic macroalgae; ** MAE: Microwave-assisted extraction of Baltic macroalgae (60 °C); EP: Extraction percentage from raw algal biomass; <LLD: Below low limit of detection.

**Table 3:** Concentration of released reducing sugars (RRS) as glucose equivalent in enzymatic extracts from *Enteromorpha prolifera* at a given control point.

	Enzyme Dose (μL) Time (h)	10	SSDD	20	SSDD	50	SSDD	100	SSDD
(Control Point)									
6 (1st)		Mean glucose concentration ± standard deviation (mg/mL)							
SSDT		0.998 ^a,b,A,c,d,e,f^ ± 0.005	A	0.969 ^g,h,i,j,k,B,l,m,n,o^ ± 0.005	b	1.02 ^p,q,r^ ± 0.01	e,f,g	1.03 ^s,C,t^ ± 0.01	i,j,B
8 (2nd)		-		-		-		-	
SSDT		1.09 ^g,u,w,D^ ± 0.01	A,a	1.08 ^x,y^ ± 0.01	b,c,d	1.20 ^a,h,s,z,^^α^^,^^β^^,^^γ^^,^^δ^^,^^ε^^,^^ζ^ ± 0.01	e,h	1.20 ^b,i,p,η,θ,ι,κ,λ,μ,ξ^ ± 0.02	i,k
10 (3rd)		a,b		c,d		a,c		b,d	
SSDT		1.03 ^z,η,E,F,π,ρ^ ± 0.03		0.976 ^u,α,θ,ς,σ,τ,υ^ ± 0.008	c	1.09 ^A,j,ι,φ,χ,ψ^ ± 0.04	h	1.08 ^k,β,ω,G^ ± 0.05	k,m
12 (4th)				e,f		e		f	
SSDT		1.06 ^B,γ,κ,H,ä^ ± 0.02		1.03 ^δ,λ,ë,ï^ ± 0.02		1.12 ^c,l,C,E,ς,ö,ø^ ± 0.00	f	1.12 ^d,m,q,F,σ,ü,ÿ^ ± 0.02	j
24 (5th)				A,B		A		B	
SSDT		0,976 ^x,ε,μ,φ,ω,ö,ü^ ± 0.024	a	0,980 ^w,ζ,ξ,χ,G,ø,ÿ^ ± 0.024	d	1.15 ^e,n,t,π,τ,H,ë^ ± 0.05	g	1.19 ^f,o,r,D,y,^^ρ^^,^^υ^^,^^ψ^^,ä,ï^ ± 0.07	B,m
6 (1st)		g,h		i,j		g,i		h,j	

SSDD: Statistically significant differences indicated between the results for the same enzyme dose at consecutive control points (extraction time); SSDT: Statistically significant differences indicated between the results for different enzyme doses at the same control point (extraction time); a, b…z; α, β…ω; ä, ë…ÿ: Pairwise indication of statistically significant differences at *p* < 0.01; A, B…H: Pairwise indication of statistically significant differences at *p* < 0.05.

**Table 4:** Total activity of Celluclast 1.5 at a given control point.

	Enzyme Dose (μL) Time (h)	10	SSDD	20	SSDD	50	SSDD	100	SSDD
(Control Point)									
		Total enzyme activity (U)							
6 (1st)		1.54 ± 0.01	a,b,c,d	1.49 ± 0.01	k,l,m,n	1.58 ± 0.01	u,v,w,x	1.59 ± 0.01	ε,ζ,η,θ
SSDT				a,b		a		b	
8 (2nd)		1.26 ± 0.01	a,e,f,g	1.24 ± 0.01	k,o,p,q	1.38 ± 0.02	u,y,z,α	1.38 ± 0.02	ε,ι,κ,λ
SSDT		c,d		e,f		c,e		d,f	
10 (3rd)		0.939 ± 0.031	b,e,h,i	0.894 *^,^** ± 0.007	l,o,r,s	0.999 ± 0.040	v,y,β,γ	0.987 ± 0.042	ζ,ι,μ,ξ
SSDT		A		g,h		A,g		h	
12 (4th)		0.796 ± 0.015	c,f,h,j	0.777 ± 0.013	m,p,r,t	0.844 * ± 0.003	w,z,β,δ	0.844 ** ± 0.012	η,κ,μ,π
SSDT				B,i		B		i	
24 (5th)		0.365 ± 0.009	d,g,i,j	0.366 ± 0.009	n,q,s,t	0.429 ± 0.018	x,α,γ,δ	0.446 ± 0.024	θ,λ,ξ,π
SSDT		C,j		D,k		C,D		j,k	
		Absolute enzyme activity (U/mL)							
6 (1st)		154 ^A,B^ ± 1		74.7 ± 0.4		31.5 ± 0.2		15.9 ± 0.1	
8 (2nd)		127 ^C^ ± 1		62.4 ± 0.5		27.8 ± 0.3		13.9 ± 0.2	
10 (3rd)		94.8 ± 3.1		45.1 ± 0.4		20.2 ± 0.8		10.0 ± 0.4	
12 (4th)		81.6 ± 1.5		39.8 ± 0.7		17.3 ± 0.1		8.66 ^A^ ± 0.12	
24 (5th)		37.6 ± 0.9		18.9 ± 0.5		8.85 ± 0.38		4.60 ^B,C^ ± 0.25	

SSDD: Statistically significant differences indicated between the results for the same enzyme dose at consecutive control points (extraction time); SSDT: Statistically significant differences indicated between the results for different enzyme doses at the same control point (extraction time); a–z, α–π: Pairwise indication of statistically significant differences at *p* < 0.01; A–D: Pairwise indication of statistically significant differences at *p* < 0.05; *,**: Pairwise indication of non-significantly different results (*p* ≥ 0.05).

**Table 5:** Methodology of *E. prolifera* hydrolysis based on literature data.

**Enzyme concentration (%, *v/v*)**	1 × 10^−2^	*
	2 × 10^−2^	*
	5 × 10^−2^	*
	9.99 × 10^−2^	[19,29]
**Biomass:medium ratio**	1 g:100 mL (each enzyme dose)	[19,29]
	4 g:200 mL (selected enzyme dose)	[21] (1 g:60 mL)
**Medium, pH**	Citrate buffer, pH 4.8	[30]
**Temperature**	50 °C	[19]
**Efficacy—enhancing action**	Rotary shaking, 200 rpm	*
**Time**	6 h	*
	8 h	[21]
	10 h	*
	12 h	[19,29]
	24 h	*

*: Variables developed for the present work.
